# Role of Metabolic Reprogramming of Long non-coding RNA in Clear Cell Renal Cell Carcinoma

**DOI:** 10.7150/jca.62683

**Published:** 2022-01-01

**Authors:** Huijie Zhang, Lei Yu, Jing Chen, Liting Liu, Xudong Yang, Hongwei Cui, Genquan Yue

**Affiliations:** 1 Clinical research center, The Affiliated Hospital of Inner Mongolia Medical University/Key Laboratory of Medical Cell Biology of Inner Mongolia Autonomous Region, Hohhot 010050, China.; 2 Department of Pharmacy, Traditional Chinese Medicine Hospital of Inner Mongolia Autonomous Region, Hohhot 010020, China.; 3 Department of Medicine, Ordos Institute of Technology,Inner Mongolia Autonomous Region, Ordos 017000, China.; 4 Department of Urology, The Affiliated Hospital of Inner Mongolia Medical University, Hohhot 010050, China.

**Keywords:** renal clear cell carcinoma, LncRNAs, metabolic reprogramming

## Abstract

Renal cell carcinoma (RCC), one of the most frequent cancers, is a "classical" malignancy characterized by metabolic reprogramming. Clear cell renal cell carcinoma (ccRCC) is its most common histopathological subtype. Long-stranded non-coding ribonucleic acids (LncRNAs) are regulatory RNA molecules with limited protein-coding capacity and evolutionary conservation. Recent studies have revealed that lncRNAs can broadly regulate the metabolic reprogramming of ccRCC and its malignant transformation. However, there are few studies on lncRNAs regulating the metabolism of ccRCC, and the specific mechanisms are unknown. Therefore, this paper summarizes the regulatory mechanisms of lncRNAs in the metabolism of ccRCC, especially in the pathways of glycolysis, mitochondrial function, glutamine and lipid metabolism, cellular mechanisms, interactions with other molecules, specific scientific and clinic implications and applications to provide a basis for early clinical diagnosis, prediction and treatment. We also discuss the clinical application and challenges of targeting lncRNAs in ccRCC metabolism.

## Introduction

Renal cell carcinoma (RCC), abbreviated as renal cancer, is highly malignant with more than 400,000 new cases per year and a global mortality rate of 2.4/100,000 1. Clear cell renal cell carcinoma (ccRCC) accounts for 70-75% of RCC 2. It has been found that ccRCC has a complex metabolic ecology containing multiple molecular mechanisms converging to alter overall cellular metabolism 3. Currently, several ccRCC metabolic reprogramming-related pathways have been identified through metabolomics and proteomics. Ninety percent of the transcribed genome can be transcribed into non-coding RNAs (ncRNAs). Based on size, ncRNAs can be classified into small noncoding RNAs and newly characterized long non-coding RNAs (LncRNAs) 4. lncRNAs are large RNA transcripts more than 200 nucleotides in length that regulate cancer cell malignancy by participating in ccRCC cell metabolic reprogramming. They regulate the malignant transformation of cancer cells and control cellular energy metabolism by participating in the "metabolic reprogramming" of ccRCC cells 5. However, the molecular characteristics and metabolic regulatory mechanisms of lncRNAs in ccRCC are still incomplete. Therefore, in this review, we elucidate the expression patterns and functions of lncRNAs in the metabolic reprogramming of ccRCC and further focus on specific pathways or mechanistic features.

## Metabolic reprogramming in tumor cells

**Figure 1:**
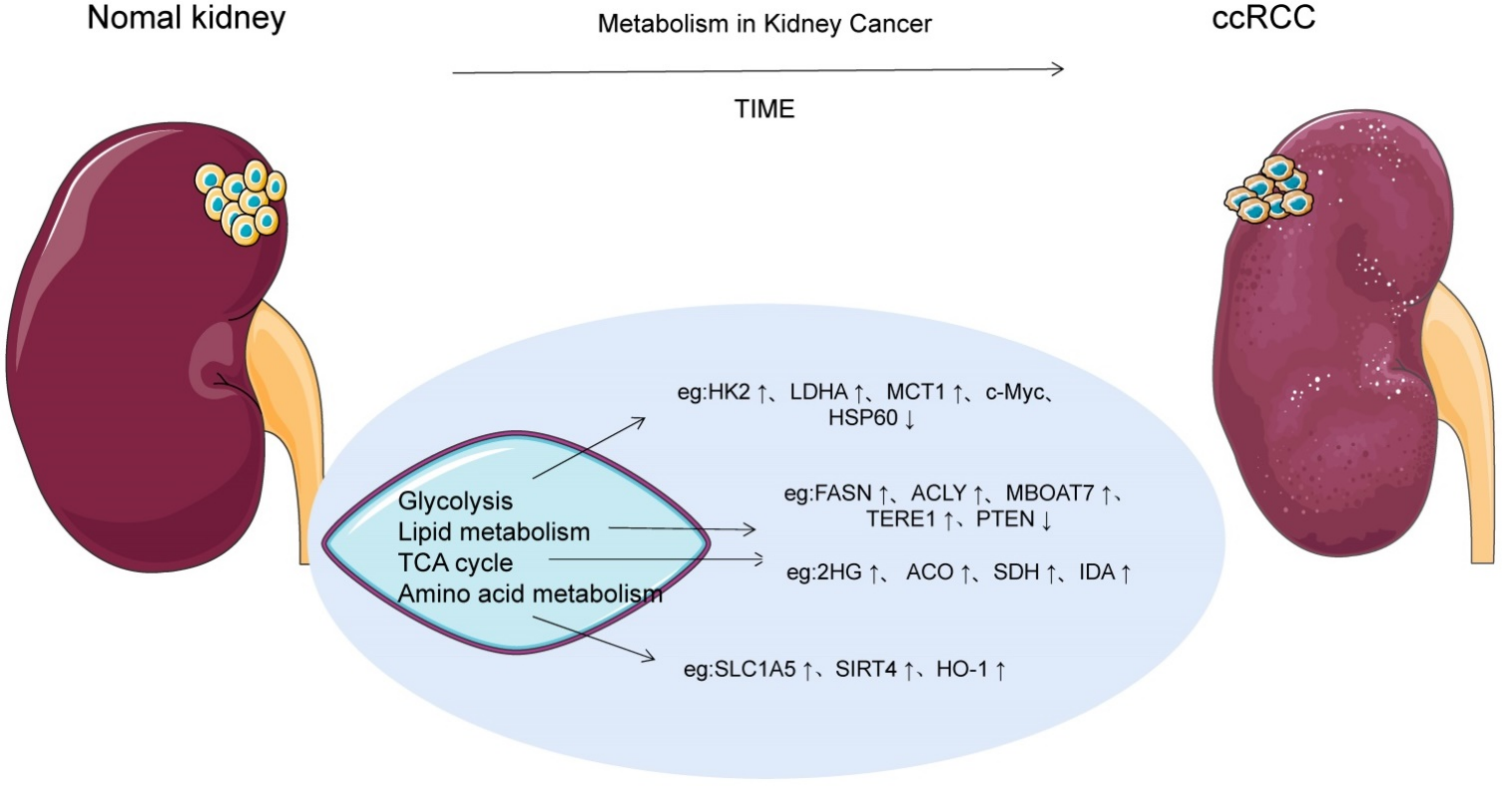
ccRCC cell metabolism. With the changes of intracellular metabolism of ccRCC, intermediates and metabolic enzymes of related pathways also showed significant changes.

Tumor cells undergo multiple metabolic changes, resulting in the accumulation of lactic acid, nitric oxide, reactive oxygen species and other by-products, which affect the composition and function of the tumor microenvironment to adapt to the nutrient-depleted microenvironment for rapid proliferation and invasion. "Metabolic reprogramming" is the change in metabolic pathways that control tumor energetics and biosynthesis. Metabolic reprogramming has long been recognized as a hallmark of many cancers, meeting the basic needs of tumor cells and increasing the level of cellular building blocks such as DNA, nucleotides, membrane components and tumor energetics molecules 3. Otto Warburg 6 first recognized aerobic glycolysis in the 1920s and argued that cancer cells meet their rapid and unlimited proliferation needs through high rates of glycolysis. And the Warburg effect has been demonstrated in a variety of tumor cell metabolisms, such as non-small cell lung cancer, breast cancer, osteosarcoma, and urinary tract tumors. In addition to the Warburg effect, the anabolic/catabolic metabolism of fatty acids and amino acids supports the supply of carbon atoms at the center of tumor cells, especially the mitochondrial functional generation that provides the energy required for rapid proliferation and maintenance of high proliferation rates. Recently, ccRCC has been considered as a metabolic disease after integrating molecular profiling studies 7. Outeiro-Pinho G et al. found that ccRCC cell metabolism is dominated by four types of macromolecular changes such as carbohydrates, lipids, amino acids and nucleic acids, which can be jointly involved in the regulation of multiple molecular mechanisms 5. It also relies on the "reflux" of glutamine metabolism and the tricarboxylic acid cycle, where reductive carboxylation occurs, allowing rapid ATP production, maintaining ccRCC energy and strictly appropriate redox status 8. In addition, with changes in intracellular metabolism of ccRCC, intermediates and metabolic enzymes of related pathways also showed significant changes, as shown in Figure 1.

### Glycolysis and TCA cycle

**Figure 2:**
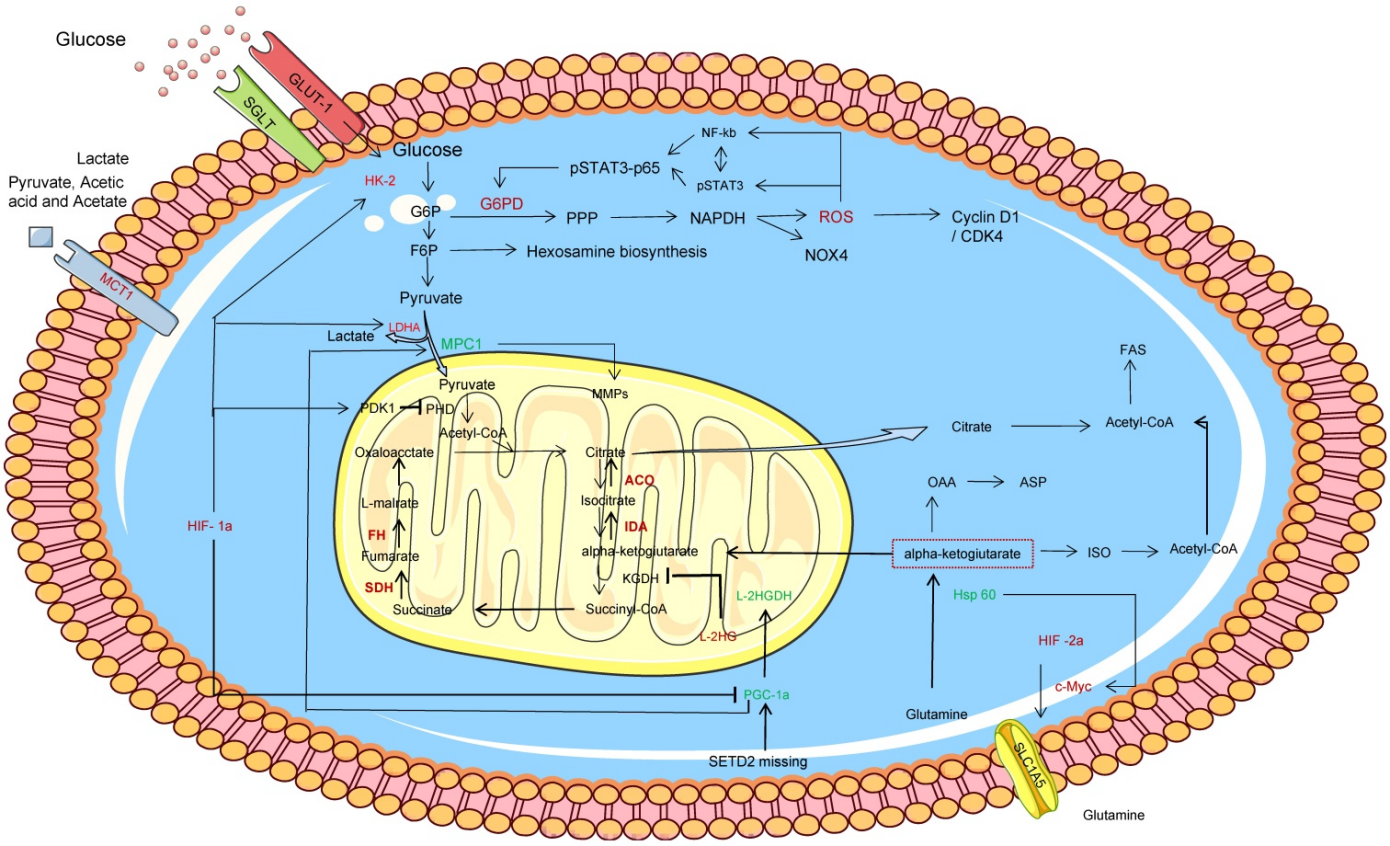
Glycolysis and the TCA cycle are involved in metabolic reprogramming in ccRCC. In this figure, the red word represents the molecule with high expression in ccRCC cells, while the green word represents the molecule with low expression in ccRCC cells. a-KG, α-ketoglutarate; GSH, glutathione; FA, fatty acid; GLUT‑1, glucose transporter 1; G6P , glucose‑6‑phosphate; HK, hexokinase; LDH-A, lactate dehydrogenase A; ROS, reactive oxygen species; SDH, succinate dehydrogenase; ASS1, Arginine succinate synthase 1; FASN, Fatty acid synthase.

The Warburg effect is one of the earliest evidences of metabolic reprogramming in cancer, and ccRCC follows the classic Warburg effect, as shown in Figure 2. HIF activity is one of the major influences that alter cell glucose input and utilization. Currently, IT is known that HIF signaling is mainly responsible for the dysregulation of six key glycolytic genes in ccRCC, such as GLUT1, HK2, AND LDHA, as well as activation of pyruvate dehydrogenase kinase (PDK) and inhibition of pyruvate dehydrogenase complex (PHD) to prevent pyruvate from being catalyzed as acetyl-CoA into the TCA cycle 9. Glucose transporter 1 (GLUT-1), sodium glucose junction transporter (SGLT), and monocarboxylic acid transporter 1 (MCT1) levels are all elevated in ccRCC tumors, and GLUT-1 is regulated by HIF-1α and increases glucose uptake 10. MCT1 is distributed in ccRCC cell membrane and promotes the uptake of L-lactic acid, pyruvate, acetic acid and acetate 10. Acetate can be metabolized to acetyl coA, which is involved in fatty acid synthesis and protein acetylation. When glucose enters ccRCC cells, it is phosphorylated to glucose-6-phosphate (G6P) by HK-2, and part of it is converted to fructose-6-phosphate (F6P) by isomerase to participate in the hexosamine biosynthesis pathway, in which glucose-6-phosphate dehydrogenase (G6PD) is modified by O-GlCNAC (OGT). As a result, the activity of G6P was enhanced through PPP, and sufficient NADPH was generated and NOX4 expression was supported, thus maintaining redox state. At the same time, G6PD promotes phosphorylation of reactive oxygen species (ROS), while ROS activation leads to over-activation or mutual activation of NF-κB and pSTAT3 signals, synergistically promoting G6PD expression, and ultimately mediated proliferation through downstream signals, such as cell cycle egg D1 regulation 11. In the other part, glucose is synthesized into pyruvate through glycolysis pathway, which is catalyzed by LDHA into lactic acid or transported to the mitochondrial inner membrane by mitochondrial pyruvate vector 1 (MPC1) to participate in the TCA cycle 12. The expression of LDHA was up-regulated by HIF-1α in ccRCC, while MPC1 was the opposite. Therefore, the antagonistic effect of LDHA and MPC1 significantly increased the production of lactic acid in ccRCC cells. The MPC1 promoter was also found to be regulated by estrogen associated receptor α (ERR-α) and peroxisome proliferator-activated receptor γ coactivator 1α (PGC-1α) 12-13. Notably, HIF signals also inhibit PGC1α^9^. Bioinformatics analysis of mRNA expression data in renal tumors showed that L2-hydroxyglutarate dehydrogenase (L2HGDH) was co-expressed with the gene encoding TCA cyclase and the gene encoding the transcription factor PGC-1α, and increased 2-hydroxyglutarate (L-2Hg) level inhibited the cyclaseα -ketoglutarate dehydrogenase. Ultimately, α-ketoglutaric acid accumulation and TCA cycle obstruction 14. On the contrary, IDH1 gene mutation results in a new activity of the isocitrate dehydrogenase (IDH) enzyme, which catalyzes the reduction of α-KG to 2HG and thus reduces the accumulation of α-KG 15. In addition, L2HG is involved in angiogenic mimicry (VM) formation in ccRCC cell lines by decreasing PHLDB2 expression or promoting activation of erK1/2 pathway 16.

Glutamine provides fuel for THE TCA cycle as another carbon source, and glutamylation and reduced carboxylation contribute significantly to the TCA cycle. Sato T et al. found that the high expression of glutamine uptake transporter solute vector family 1 member 5 (SLC1A5) increased the uptake of glutamine in ccRCC cells and catalyzed α -ketoglutaric acid for TCA recycling 17. Lactate derived pyruvate stabilizes HIF-1α and HIF-2α by inhibiting PHD, and HIF-2α promotes MYC transcriptional activity, such as SLC1A5. Zhang C et al. found that methyltransferase-like 14 (METTL14) was low expressed in ccRCC and was related to differential genes such as NR1D1, BPTF and BMP2, among which BPTF and MYC interact to promote glycolysis flow^18^. HSP60 is the main mitochondrial chaperone that maintains mitochondrial protein stability. Teng R et al 19 found that HSP60 was significantly down-regulated in ccRCC, which disrupted the mitochondrial protein stability in ccRCC cells and enhanced the flow of Gln→αKG→OAA→Asp and Gln→αKG→ISO→ acetyl-CoA. Lead to increased de novo nucleotide synthesis and lipid synthesis.

### Lipid metabolism

**Figure 3:**
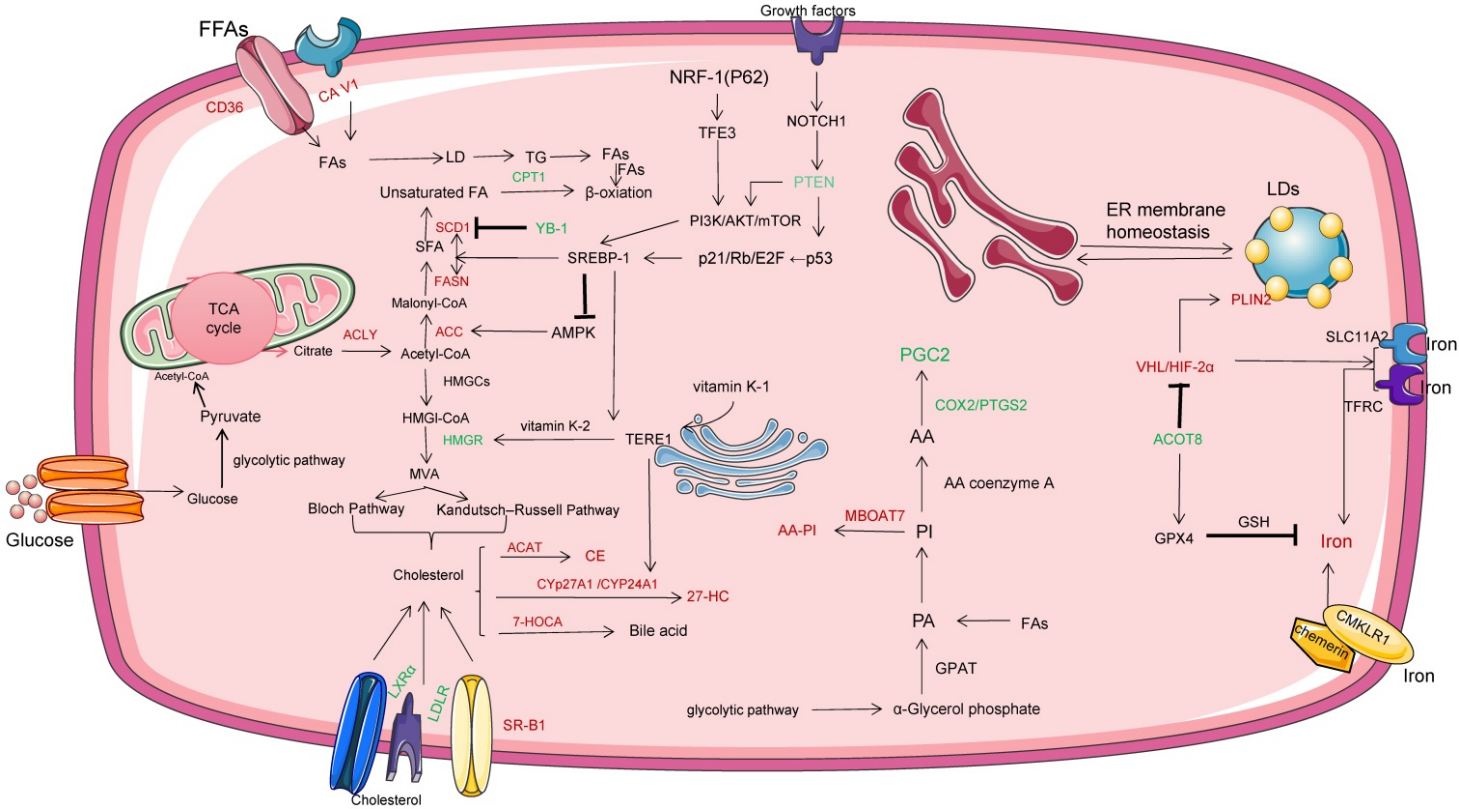
Lipid metabolism reengineering involves several aspects, such as increased lipid uptake , FAS and FAO. In this figure, the red word represents the molecule with high expression in ccRCC cells, while the green word represents the molecule with low expression in ccRCC cells. HMGCR, 3-hydroxy-3-methylglutaryl-coenzyme A reductase; AA-PI, arachidonic acid containing PI; LPI, lysophosphatidylinositol; MBOAT7, Membrane bound O-acyltransferase domain 7; ACOT8, acyl-coenzyme A thioesterases; PIPs, phosphatidylinositol phosphate; ER, endoplasmic reticulum; LDs, lipid droplets; CPT1A, carnitine palmityl transferase 1A; PLIN2, Perisolipoprotein 2; HIF, hypoxia-inducible factor; MUFA, single-chain unsaturated fatty acids; YB-1, Y box binding protein 1; LXRα,liver X receptor α; FASN, atty acid synthase; ACC, acetyl-CoA carboxylase; SCD1, stearoyl-coA desaturase-1; SR-B1, scavulant receptor Class B type 1; CA V1; cellulin 1.

Lipid metabolism reengineering involves several aspects, such as increased lipid uptake, de novo fatty acid synthesis (FAS), and FAO, as shown in Figure 3. Lipids, including triglycerides, phospholipids, sphingolipids, and cholesterol, are used as energy sources, cell membrane components, and metabolized to produce molecular precursors involved in a variety of biological processes. FAs is an essential component of all biofilm lipids, a second messenger of signals and an important energy source. In ccRCC, FAs is further modified by lengthening enzyme or desaturase to form more complex FAs, which promotes the rapid proliferation and invasiveness of tumors. CD36, also known as fatty acid translocation enzyme (FAT), scavulant receptor Class B type 1(SR-B1), and cellulin 1(CA V1), as lipid and cholesterol receptors, are increased in ccRCC compared with normal tissues, while the transcription levels of low density lipoprotein receptor (LDLR) and liver X receptor α (LXRα) are decreased 20. Circulating free fatty acids (FFA) are lipolysed and absorbed by cells via lipid receptors CD36 or CA V1. CcRCC cells develop FAS mechanisms by increasing the activity of key adipogenic enzymes, such as citrate lyase (ACLY), acetyl-CoA carboxylase (ACC), fatty acid synthase (FASN) and stearoyl-coA desaturase-1 (SCD1) 21. ACC catalyzes the production of malonyl-coA for the conversion of citric acid and acetate to acetyl-coA. FASN is upregulated in most ccRCC tissues and is the main synthase of long chain fatty acids, which contributes to the formation of lipid droplets and is also involved in carboxylic acid binding and vitamin binding. FASN associated adipogenesis depends on the activity and/or expression of important oncogenes and tumor suppressors, such as p53 and MYC 22. P53 inhibits SREBP1 expression through the P21 (cyclin-dependent kinase inhibitor 1A) /Rb/E2F transcription factor pathway. FASN and SREBP-1 enhance FAS and inhibit AMPK phosphorylation to stabilize ACC1 protein. In ccRCC cells, the tumor suppressor gene PTEN activates the PI3K cascade via p53-mediated transactivation, while NOTCH1 cascade regulation increases the level of PTEN and induces increased expression of participating lipid biosynthases, including ACLY, FASN and ACC. However, it has recently been found that accumulation of NADH leads to PTEN inactivation 23,24.

Y box binding protein 1 (YB-1) plays a role as DNA and RNA binding protein, promoting or inhibiting the expression of target genes. Jeffords E et al. found that YB-1 is very sensitive to single-chain unsaturated fatty acids 25. The binding of YB-1 to the SCD1 promoter reduces the level of endogenous MUFA in cells and prevents the toxic accumulation of saturated fatty acids. It is speculated that there is a potential feedback mechanism between the levels of fatty acids in ccRCC by YB-1.

The cancer phenotype of ccRCC is associated with hypoxia-inducible factor (HIF) signaling and intracellular lipid droplet (LDs) accumulation. For example, HIF inhibits the expression of carnitine palmityl transferase 1A (CPT1A) and reduces the transport of fatty acids to mitochondria. Besides reducing acetyl coA production through pyruvate decarboxylation, it also reduces acetyl coA production through β oxidation, thus forcing lipid droplet storage 26. It is currently believed that excess lipids (including excess FAs and cholesterol) in ccRCC cells reside in the core of LDs as neutral, inert biomolecules. Perisolipoprotein 2 (PLIN2), a lipid droplet coat protein, is significantly higher in ccRCC than in normal cortex culture, and HIF-2α regulates PLin2-dependent lipid storage, thereby inhibiting cytotoxic er stress response 27. However, how PLIN2 regulates hiF-2α downstream lipid metabolism and storage has not been identified. LDs function is also associated with the endoplasmic reticulum (ER), which facilitates the exchange of lipids and proteins between organelles via transient membrane Bridges. In addition, overexpression of HIF-α and activation of TFRC and SLC11A2 drivers contribute to iron uptake and assist the activities of various metabolic enzymes in ccRCC cells, including catalase, desatase and actinase, revealing a possible new mechanism of interaction between VHL/HIF-α axis and ccRCC iron metabolism 28. Xu CL et al.found the potential role of acyl-coa thiesterase (ACOT8) in oxidative phosphorylation regulation of ccRCC, in which ACOTs was significantly down-regulated and showed that ACOT8 upregulated the expression of iron death suppressor genes, such as glutathione peroxidase 4(GPX4) 29. Promotes glutathione (GSH) and reduces the production of lipid peroxides to play a protective role.

Phosphatidylinositol (PI) and the closely related phosphatidylinositol phosphate (PIPs) are essential for eukaryotic life. Lucarelli G et al. study found that the lipid characteristics of ccRCC included the increase of phosphatidylinositol rich in arachidonic acid 20. Membrane bound O-acyltransferase domain 7 (MBOAT7), as an acyltransferase, can selectively esteridate lysophosphatidylinositol (LPI) lipids into arachidonic acid-coA, forming arachidonic acid containing PI (AA-PI) in the inner lobule of ccRCC membrane 30. Notably, MBOAT7 differs from other lysophosphatidyltransferases in that it only diversifies the fatty acid composition of membrane PI species. In addition, MBOAT7 deletion of ccRCC cells reduced growth factor-driven MAPK activation.

As an important component of cell membrane, cholesterol changes the biophysical properties of cell membrane fluidity and affects various biochemical functions. It has been reported that the cholesterol level of about 70% of tumors in primary ccRCC samples is at least two times higher than that of benign kidney tissue 31. Context HDL is synthesized by apolipoprotein A1 through ABCA1, and is mediated by Scavenger receptor Class B (Type 1) and catalyized by ACAT, which promotes Cholesterolester(CE) in ccRCC. CE for the synthesis of steroid hormones, vitamins and bile acids 32. It is noteworthy that SR-B1 is almost not expressed in normal kidney tissues 33. Cholesterol is synthesized by the mevalic acid pathway and reduced to mevalic acid by its rate-limiting enzyme, 3-hydroxy-3-methylglutaryl-coenzyme A reductase (HMGCR). It has been reported that the expression of HMGCR is affected by multiple factors, such as PI3K/AKT signal, RAS/MAPK signal, SREBP2 and SREBP cleavage activating protein regulatory proteins, which mediate cholesterol biosynthesis to maintain cholesterol homeostasis 34. Meanwhile, hypoxic of ccRCC cells induced HIF-1A transcription in ccRCC cells and increased HMGCR level. In addition, epoxide sterol as a metabolic intermediate is speculated as a potential new metabolic pathway to transform cholesterol in kidney into cholesterols. Recently, Fredericks WJ et al. proposed that Ectopic expression of the TERE1 (UBIAD1) mediates the metabolism of vitamin K2 in ccRCC cells 35. Such as driving fumarate reductase and causing succinic acid to rise or interacting with HMGR and SOAT1 to reduce cholesterol synthesis and storage. TERE1 is a tumor suppressor consisting of 338 amino acid residues. TERE1 increases the expression of cytochrome CYP24A1, which is normally lost in renal cell carcinoma and is required for vitamin D3 transformation.

### Amino acid metabolism

**Figure 4:**
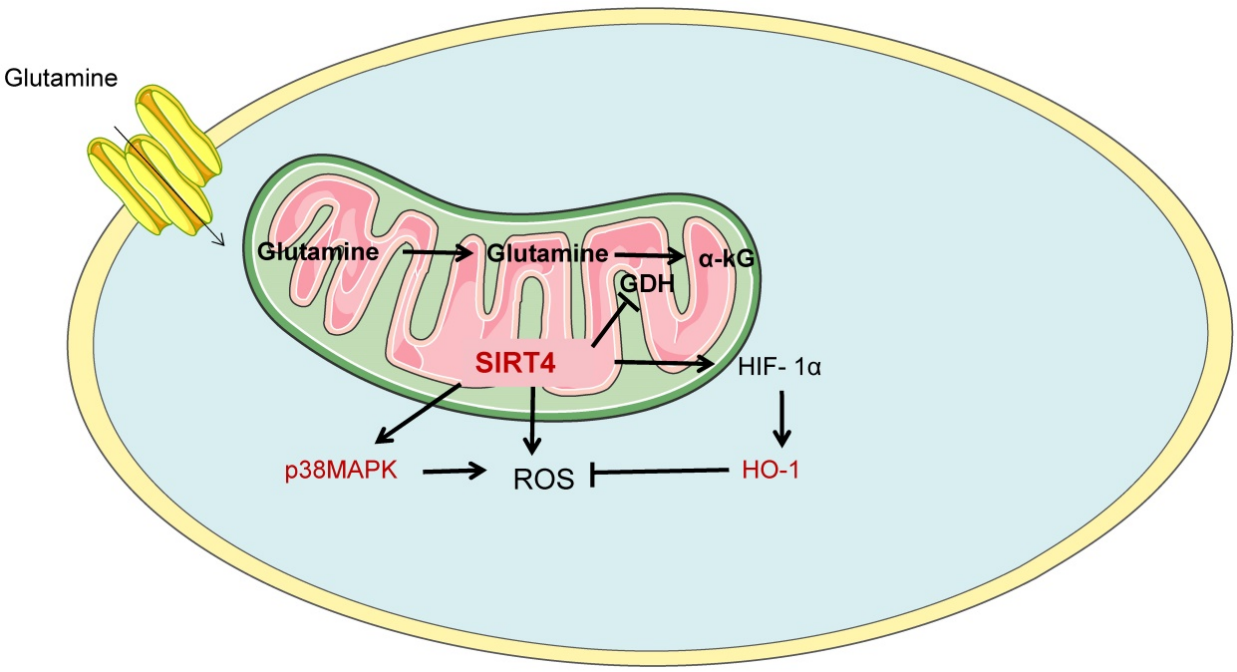
SIRT4 is involved in glutamine metabolism. Glutamine metabolism is another important mode of energy metabolism in ccRCC cells. SIRT4, as a novel molecule, has been shown to be involved in alternate metabolism of glutamine and regulation of tumor microenvironment. SIRT4, Sirtuin 4; HO-1, Heme oxygenase-1.

Glutamine metabolism is another important mode of energy metabolism in ccRCC cells. Glutamine is by far the most abundant amino acid in plasma and is therefore a rich cellular fuel. In the cytoplasm, glutamine is introduced into the cell via the glutamine transporter ASCT2, which is subsequently converted to glutamate by glutamine dehydrogenase or transaminase and converted to α-KG (intermediate products of the TCA cycle) in combination with the production of NADH, NADPH, ammonium, and other non-essential amino acids. And provide precursors for the synthesis of amino acids, nucleotides and fatty acids, such as citrate and oxaloacetate. In addition, glutathione is a major factor in alleviating intracellular REDOX stress. Tong Y et al. believed that mitochondrial protein Sirtuin 4 (SIRT4), as a new molecule, was proved to be related to alternate metabolism of glutamine and regulation of tumor microenvironment^36^. SIRT4 is an unstudied member of the Sirtuin family. In ccRCC cells, SIRT4 promotes apoptosis by enhancing intracellular reactive oxygen species (ROS). Meanwhile, down-regulation of SIRT4 promoted the up-regulation of heme oxygenase-1 (HO-1) in hypoxic cells, thereby counteracting the promoting effect of SIRT4 on ROS accumulation and apoptosis. Moreover, SIRT4 regulates ROS and HO-1 expression through phosphorylation of Akt and P38MAPK, as shown in Figure 4.

## lncRNAs biological functions

**Figure 5:**
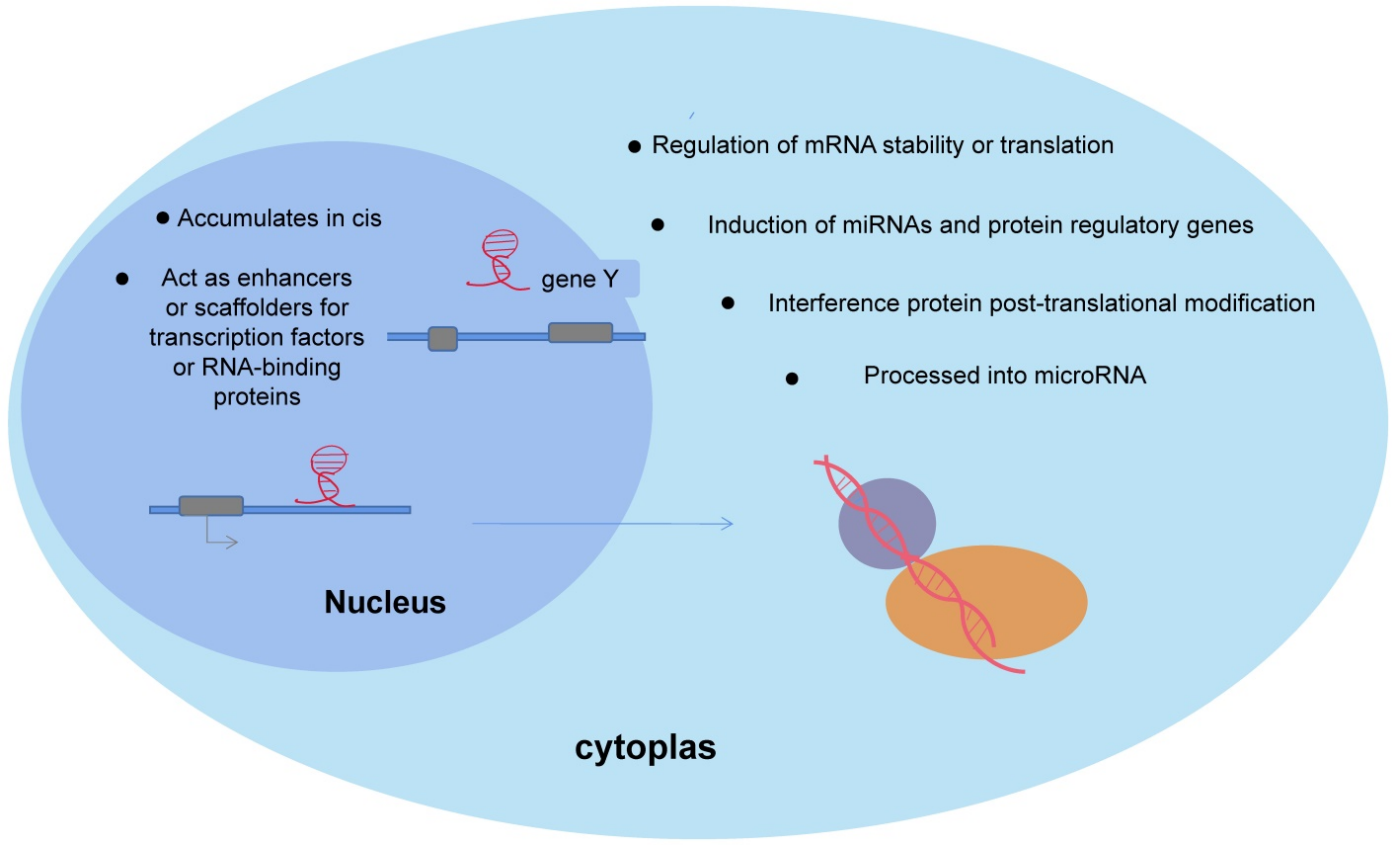
Biological function of lncRNAs. In the nucleus, the gene specificity of lncRNAs enables their relocalization at the synthesis site to affect gene regulation or transcription, and thus regulate the expression of adjacent genes and play a cis-regulatory role. In cytoplasm, lncRNAs decay mRNA and regulate mRNA stability or translation, induction of miRNAs and proteins and influence gene regulation.

lncRNAs are important isoforms of ncRNAs, and lack protein-coding ability. More than 68% of the genes expressed in the human transcriptome are transcribed to non-coding regions. Based on the location of protein-coding genes, lncRNAs are classified as: sence, antisense, bidirectional, intronic and intergenic transcripts. Extensive studies have shown that lncRNAs are aberrantly expressed in many human cancers, control cellular energy metabolism, and have integrative functions in cancer cell genesis and development 37. A recent study using single-molecule RNA fluorescence in situ hybridization showed that LncRNAs function in relation to their unique subcellular localization 38, as shown in Figure 5. First, LncRNAs, as important regulators of nuclear function, exhibit different patterns of nuclear localization. In the nucleus, the gene-specific nature of lncRNAs allows them to re-localize at synthetic sites to influence gene regulation or transcription, which in turn regulates the expression of neighboring genes, acting as cis-regulators 39, as in HOTAIR. Meanwhile, lncRNAs modify gene expression by directly interacting with transcription factors or RNA-binding proteins, acting as enhancers or scaffolds 40. However in the cytoplasm, LncRNAs, as competitive endogenous RNA (ceRNA), decay microRNAs and regulate the stability or translation of mRNAs, or compete with microRNAs for binding mRNAs 4. Moreover, lncRNAs can affect gene regulation by inducing miRNAs and proteins. In addition, lncRNAs were found to interfere with protein post-translational modifications, leading to aberrant signaling 41, such as lncRNA MEG3 and ST3β-galactoside alpha2, 3 sialyltransferase 1 (ST3Gal1) signaling interactions and interferes with the phosphorylation of the epidermal growth factor receptor (EGFR). Fedorko M et al. found that LncRNAs are involved in chromatin, protein and RNAs interactions in the nucleus/cytoplasm of ccRCC cells to cis or trans manner to regulate genomic expression and post-transcriptional regulation, altering cellular physiological and pathological kinetics such as energy metabolism, lipid synthesis, inflammation, cell differentiation and cancer development^42^.

## Dysregulated LncRNAs in ccRCC cells

In recent years, with the continuous development of small sample sequence analysis method for determination of the genetic progress, one of the most commonly used experimental methods for microarray analysis and small sample experiment of chromatin immune coprecipitation sequencing (Chip-seq). Chip-seq was sequenced in small samples. If some lncrnas were found to be obviously maladjusted, the significance of chIP-SeQ in large samples was proved to be the same by qPCR. Some of these abnormally expressed LncRNAs may be used as biomarkers for diagnosis or prognosis. More than 100,000 lncrnas with large differences between renal cancer tissues and para-cancer tissues were identified by whole-genome sequencing 43. Qi-Dong X et al. used the Cancer Genome Atlas (TCGA) and the International Cancer Genome Consortium (ICGC) database to download LncRNAs expression data and corresponding clinical information of 619 ccRCC patients 44. Multivariate Cox regression was used to establish a risk model, and it was found that lncRNAs expression and risk score were significantly correlated with the survival rate of ccRCC patients (P<0.001). In the TCGA validation queue, the area under the synthesis curve (AUC) was 0.905. Whether lncRNA has specific molecular mechanism in renal cancer tissue and cell metabolism remains unclear. However, it has been suggested that this specific mechanism may be related to the androgen receptor (AR)/ hypoxia-inducible factor-2 a(HIF-2A)/MYC pathway.

## LncRNAs are involved in the "metabolic reprogramming" of ccRCC cells

**Figure 6:**
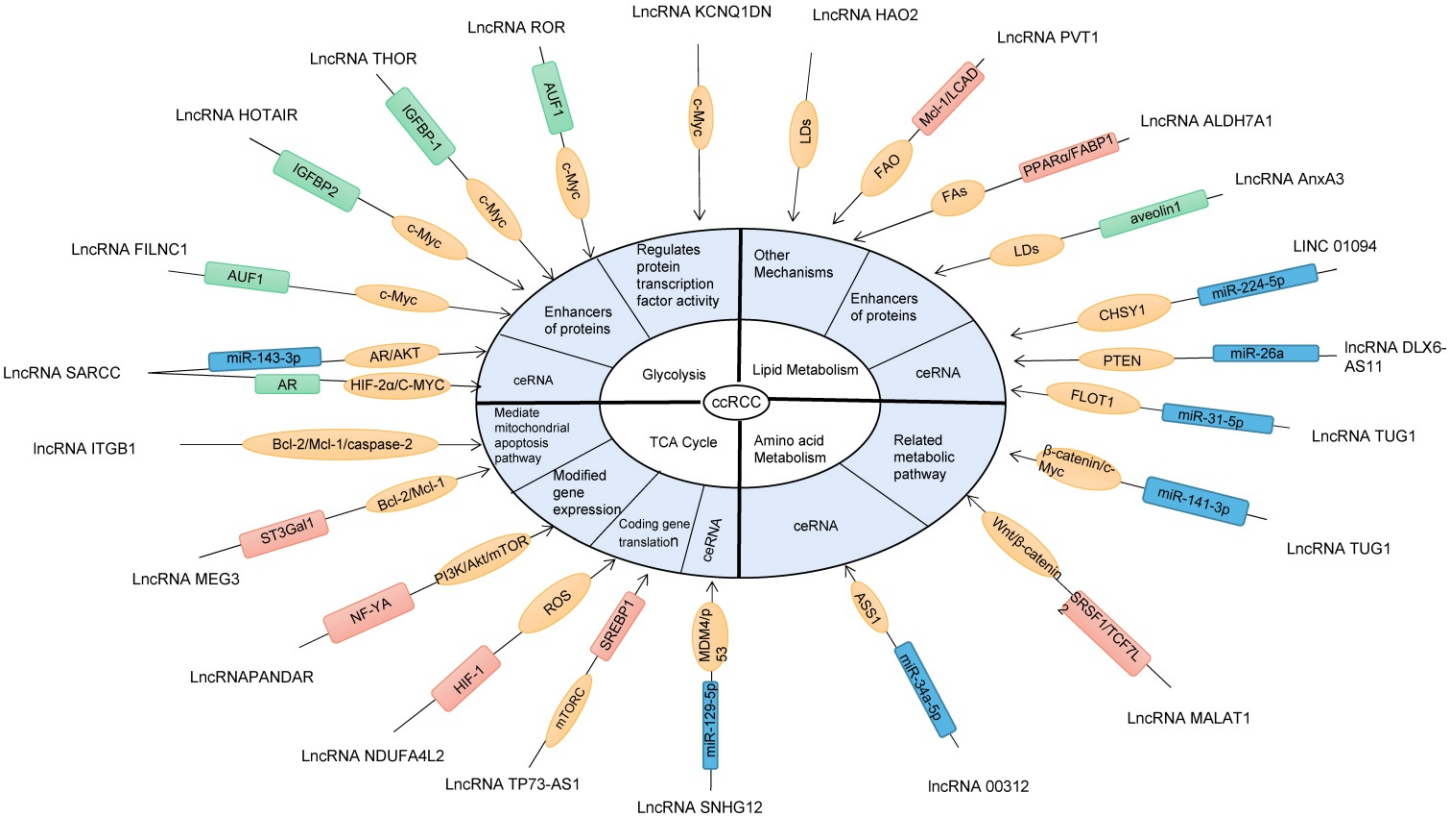
IncRNAs is involved in different pathways or mechanisms of ccRCC metabolic reprogramming. MultipleIncRNAs participate in the metabolic reprogramming pathway of ccRCC and regulate the biological behavior of ccRCC cells. Metabolic reprogramming of ccRCC includes glycolysis, TCA cycle, lipid metabolism and amino acid metabolism. In the figure, the blue boxes represent IncRNAs acting as ceRNA, the green boxes represent proteins interacting with IncRNAs (enhancers) and the pink boxes represent other molecules interacting with IncRNAs. ROS, reactive oxygen species; ASS1, Arginine succinate synthase 1; FA, fatty acid; MICU1, Mitochondrial calcium uptake 1; mTORC1, Rapamycin complex; IGFBP, Insulin-like growth factor-binding protein-1; VLCAD, Very long chain acyl CoA dehydrogenase.

LncRNAs target the "metabolic reprogramming" pathway and related metabolic enzymes in ccRCC through multiple mechanisms, as shown in Figure 6 and Table 1. The mechanism studies published so far show that most of the malregulated lncrnas in ccRCC play their biological functions through ceRNA. Meanwhile, lncrnas also act as miRNAs or protein enhancers to affect gene regulation, or interfere with post-translational modification of proteins, leading to abnormal signal transduction and inducing malignant transformation of ccRCC.

## LncRNAs and Glycolysis

### LncRNAs as ceRNA

LncRNAs, as a ceRNA, interact with mirnas and affect miRNA activity through isolation, thereby indirectly mediating post-transcriptional upregulation of tumor miRNA target mRNA. In ccRCC, most lncRNAs discovered so far are considered as miRNA sponges or decoys 40. Recent studies have shown that Myc-dependent metabolic reprogramming is a key factor in tumorigenesis and development 45. C-myc is an important member of the Myc gene family and plays an important role in many biological processes, including cell cycle, cell proliferation, apoptosis and cell metabolism. It has been reported that c-MYC is significantly upregulated in ccRCC and can directly regulate the expression of glucose metabolism genes or induce glycoly-related metabolic enzymes to increase glucose input and synthesis, such as GLUTs, HK2, phosphoglucose isomerase, phosphofructose kinase, glyceraldehyde-3-phosphate dehydrogenase, phosphoglycerate kinase and enolase 45. C-Myc was also observed by Gomes AS et al 46 to upregulate lactate dehydrogenase A expression to produce NAD+, which in turn maintains a high flow of glycolysis. It has been reported that lncRNAs may promote transcription of their host genes or protect their homologous mrnas from mirNA-mediated degradation by inhibiting miRNA activity (as ceRNA). For example, LncRNA SARCC (Suppressing Androgen Receptor in Renal Cell Carcinoma) acts as ceRNA to isolate Mir-143-3p expression. Inhibition of unstable androgen receptor (AR) protein function inhibits downstream signaling, including AKT, MMP-13, K-RAS, and P-ERK 47. In addition, lncRNA SARCC can also inhibit the hypoxia cell cycle progression of VHL mutation in RCC cells, and inhibit AR/HIF-2α/C-MYC signal through physical binding and de-stabilizing AR protein, thereby post-transcriptional regulation of AR to form a negative feedback regulatory mechanism 48.

### LncRNAs regulate protein transcription factor activity

LncRNAs mediate cell cycle arrest and apoptosis by regulating the expression of cyclin-related proteins (such as cyclin D1, p53 and P16) and apoptosis-related proteins (such as Bax and Bcl-2). LncRNA KCNQ1DN is mainly located on chromosome CHR11P15.5. YANG et al. found that KCNQ1DN was significantly reduced in ccRCC tissues and cell lines, and gene analysis showed that KCNQ1DN could affect downstream coding genes, such as GLUTs and HK2, by inhibiting the transcriptional activity of c-MyC gene promoters, thus obstructing glucose intake, inhibiting glycolysis pathway and reducing energy supply 49. Moreover, cyclin D1 is an important target of C-myC in cell cycle, and KCNQ1DN further upregulates cyclin D1 in RCC cells and induces RCC cell cycle arrest.

### LncRNAs act as enhancers of proteins

As structural components of protein complexes, lncRNAs may interact with specific proteins and enhance their function. It's worth noting that, lncRNA ROR promotes c-mRNA stabilization, inhibits p53 expression, increases c-Myc expression, induces glycolysis in ccRCC cells and thus promotes their proliferation by binding to hnRNP I and AU-rich element RNA binding factor 1 (AUF1)^50^-^51^. However,LncRNA FILNC1 (FoxO-induced long non-coding RNA 1)inhibits c-Myc gene translation by chelating AUF1 and hinders glucose metabolism gene regulation in ccRCC cells 52.

Insulin-like growth factor 2 gene-binding protein-1 (IGFBP) is a member of the superfamily of homologous proteins responsible for regulating the biological activity of insulin-like growth factor (IGF). According to the initial studies related to IGFBP, IGFBP-7 was found to be an independent candidate biomarker for early detection of acute kidney injury with high sensitivity and specificity 53. Another study showed that IGFBP2 is associated with the pathogenesis of metabolic diseases or cancer and plays a key role in regulating cellular biological processes, such as proliferation 54. It was also found that IGFBP-1 enhanced cellular antioxidant activity, downregulated the expression of Caspase3 and BCL2-Associated X (Bax) and upregulated the expression of anti-apoptotic gene Bcl-2 in ccRCC 55. It has been recently reported that LncRNA THOR directly binds to IGFBP-1 to induce ATP production and increase the transcription levels of HK2, phosphoinositol dependent protein kinase 1 and the transcription level of Myc 56, and regulates the genetic stability of key oncogenes, while promoting ccRCC proliferation. Meanwhile, Katayama H et al. analyzed the correlation between HOTAIR expression and clinical features in ccRCC and found that HOTAIR upregulates its downstream molecule IGFBP2 expression, induces glycolytic gene expression, and maintains a high flow of glycolysis in ccRCC cells, which correlates with their proliferative and migratory capacity 57.

## LncRNAs and mitochondrial dynamics

Mitochondria are at the center of many biochemical processes and are involved in their fusion or division, affecting mitochondrial shape, distribution and function. It has been found that LncRNAs regulate mitochondrial dynamics 58, such as Oxidative phosphorylation (OXPHOS), TCA cycle, intracellular calcium homeostasis, and synthesis of cytosolic biological precursors such as amino acids, nucleotides, lipids and NADPH.

### LncRNAs as ceRNA

Many LncRNAs contain a variety of types and numbers of miRNA binding sites that specifically bind mirnas, thereby reducing miRNA activity and upregulating miRNA target gene expression. P53 is a well-known tumor suppressor gene. More than 50% of malignancies show p53 mutations. Murine double minute4 (MDM4) encodes a nuclear protein that is an important regulator upstream of p53, which inhibits p53 by binding to the transcriptional activation domain and significantly suppresses the oxidative phosphorylation pathway. Therefore, MDM4 can act as an anticancer target to inhibit the trans-activation and apoptosis-inducing functions of p53. Wu Z et al.further found that LncSNHG12(small nucleolar RNA host gene 12), as a ceRNA secreting Mir-129-5P, regulates MDM4/p53 axis signaling pathway, mediates changes in mitochondrial energy metabolism, inhibits mitochondrial respiration and plays a role in regulating the development of ccRCC 59.

### LncRNAs encode gene translation

Rapamycin complex 1 (mTORC1) controls cellular metabolism by regulating the translation and transcription of metabolic genes, such as sterol regulatory element binding protein 1/2 (SREBP1/2) and HIF- 1α. mTORC1 has been shown to regulate the translation of nuclear mitochondrial genes and increase mitochondrial ATP production 60. Liu G et al.found that LncRNA TP73-AS1 mediates mTORC signal, encodes the translation of nuclear mitochondrial protein SREBP1, and generates ATP to promote ccRCC cell proliferation and inhibit apoptosis 61. Meanwhile, mTORC1 upregulation affects the ccRCC tricarboxylic acid cycle, leading to downregulation of succinate and accumulation of ferredoxin, further inducing its malignant transformation 62. IncRNA NDUFA4L2 (NADH dehydrogenase [ubiquinone] 1 alpha subcomplex, 4-like 2), a regulatory protein encoding a HIF-1 target gene that reduces mitochondrial oxygen consumption 63. Tello D et al. showed that NDUFA4L2 is highly upregulated in ccRCC tissues, controlling mitochondrial growth while counteracting the excessive production of ROS due to mitochondrial respiratory chain damage in ccRCC cells, and It increases the level of cellular antioxidant and makes ccRCC cells stronger and more aggressive 64. Moreover, NDUFA4L2 predicts ccRCC behavior, and abnormal NDUFA4L2 expression correlates with its risk of disease progression and death 63.

### LncRNAs and transcription factors modify gene expression

LncRNA PANDAR (Promoter of CDKN1 antisense DNA damage activated RNA) induces in p53-dependent mode interacts with the transcription factor NF-YA to repress the expression of pro-apoptotic genes, such as promoting the expression of Bcl-2 and McL-1, and down-regulating the expression of Bax, thereby inhibiting the PI3K/Akt/mTOR pathway leading to the proliferation and invasion of ccRCC cells 65. Meanwhile, LncRNA PANDAR can be used as an independent predictor of overall survival in ccRCC.

### LncRNAs mediate mitochondrial apoptosis pathway

MCL-1 is a member of the anti-apoptotic BCL-2 family with a short half-life and one of the most highly amplified genes in cancer. Mcl-1 may be a key factor in the control of apoptosis, regulating the early events of the associated cascade response leading to cytochrome c release. Mcl-1 exhibits different functions at different mitochondrial locations. At the outer mitochondrial membrane, the MCL-1 isoform antagonizes apoptosis like other anti-apoptotic BCL-2 molecules, whereas the amino-terminal isoform of MCL-1 imported into the mitochondrial matrix promotes normal mitochondrial fusion, ATP production, membrane potential, respiration, cristae ultrastructure and oligomeric ATP synthase activity 66. LncRNA MEG3(maternally expressed gene 3), a tumor suppressor, has been shown to be involved in the development of cancer 67-68. And Gong A et al. found that IncRNA MEG3 mediates ST3Gal1 to regulate EGFR phosphorylation, which will downregulate Bcl-2 and prcaspase-2 expression,upregulate caspase-2 and cytochrome c release^69^ , leading to mitochondrial dysfunction and inducing apoptosis in ccRCC cells. It is noteworthy that HOTAIR not only participates in glycolysis of ccRCC cells, but also mediates mitochondrial apoptosis. IncRNA HOTAIR induces mitochondrial calcium uptake 1 (MICU1)-dependent death in ccRCC cells by modulating mitochondria-related cell death pathways, such as Bcl-2, BAX and cytochrome c, and altering mitochondrial membrane potential 70-71. The expression level of lncRNA ITGB1, as an oncogenic gene, was significantly higher in ccRCC than in neighboring specimens and was closely associated with the survival of ccRCC patients. However, the exact role of ITGB1 in ccRCC remains unclear. As studied by Zheng XL et al. Mcl-1 expression in ccRCC tissues was positively correlated with ITGB1 expression, promoting normal mitochondrial fusion, rapid ATP production, supporting ccRCC energy supply, and promoting tumorigenesis in ccRCC 72.

## LncRNAs and lipid metabolism

### LncRNAs as ceRNA

Since more than half of human mRNAs are estimated to be conserved miRNA targets, LncRNAs are thought to play a broad role by regulating gene expression, as shown in a study by Xu H et al. LINC01094 upregulates solute vector family 2 and promotes glucose transporter member 1 (SLC2A) to regulate glycolysis flow and proliferation and apoptosis of ccRCC by targeting mir-184 73. Meanwhile, Jiang Y et al. reported that transcription factor FOXM1 activated LINC01094 and showed that LINC01094 acts as a molecular sponge for Mir-224-5p, activates targeted mRNA of Mir-224-5p such as CHSY1, and regulates glycolysis flow and biological process of ccRCC 74. In addition, LncRNAs inhibit miRNA activity. For example, Lv D et al. revealed that Taurine- Upregulated gene 1 (TUG1) is a 7.1-KB lncRNA located on chromosome 22q12, as ceRNA to isolate Mir-31-5p and promote flotillin 1 (FLOT1) to participate in cell membrane structure and mediate intercellular signal transduction 75. FLOT1 is a lipid raft marker protein, and its n-terminal residues at position 1 to 185 constitute an SPFH (Stomatin, Prohibitin, Flotillin, Hflk/C) domain, which is palmitoylated at C34 site and associated with its localization in lipid raft 76. In addition, LOt-1 interacts with the mitogen-activated protein kinase (MAPK) cascade signaling pathway components and acts as a scaffold protein for MAPK. Phosphatase and Tensin Homolog deleted on Chromosome 10 (PTEN) is a dual Phosphatase with protein and lipid Phosphatase activities. Mutations of PTEN in ccRCC are rare, but PTEN expression is low in most ccRCC. Meanwhile, PTEN is a direct target of Mir-26a in renal cell carcinoma. LncRNA DLX6-AS11, as a molecular sponge of Mir-26a, negatively controls THE PI3K-Akt cascade signal through Mir-26a /PTEN axis, and indirectly regulates the lipid metabolism of ccRCC cells by regulating the activities of FASN, ACC1 and SCD1, thereby affecting a variety of biological processes of renal cancer cells 77.

### LncRNAs act as enhancers of proteins

The phospholipid-binding protein Annexin A3 (AnxA3), as a LncRNA, was found to be a key factor in initiating ccRCC adipocyte differentiation and showed differential expression of two isoforms, 36 kDa and 33 kDa. The AnxA3 isoform protein was not only present in nucleated cells but also in the purified membrane fraction of ccRCC cells. The data suggest that the 36 kDa type AnxA3 silencing increases lipid storage and can act as a negative regulator of lipid storage in ccRCC cells 78. Moreover, 36 kDa ANXA3 negatively regulates lipid storage in CCRCC cells through an aveolin1-dependent endocytosis that interferes with vesicle transport involved in lipid absorption and accumulation 78.

### Other mechanisms

Hydroxy acid oxidase 2 (HAO2) belongs to the 2-hydroxy acid oxidase gene family and encodes a peroxisomal protein with 2-hydroxy acid oxidase activity. LncRNA HAO2 is mainly expressed in basic amino acids and oxidizes long-chain 2-hydroxy acids to keto acids to generate hydrogen peroxide. Xiao W et al. found that HAO2 levels of correlated with the genomic of neutral lipid catabolic processes, metabolic processes and lipid oxidation, and suggested that HAO2 eliminates lipid droplet accumulation in ccRCC cells, promotes intracellular lipid metabolic processes, inhibits extracellular matrix transformation and promotes ccRCC development 79.

Peroxisome proliferator activated receptor - α (PPARα) is a major transcriptional regulator of adipogenesis. It was found that LncRNA ALDH7A1 metabolizes lipid-active aldehydes through PPAR signaling and protects ccRCC cells from oxidative stress 80. Meanwhile, LncRNA ALDH7A1 downregulates glycerophosphorylcholine and phosphorylcholine levels, inhibits fatty acid (FAs) synthesis, and affects ccRCC cell membrane formation. In addition, fatty acid binding protein (FABP) is a key central regulator of fatty acid metabolism. Wu G et al. showed that FABP1 is co-expressed with PPARα and can synergistically mediate the oxidation of FAs and hinder ccRCC occurrence and development 81. TCGA cohort studies have shown that LncRNA PVT1(Plasmacytoma variant translocation 1) is upregulated in ccRCC and correlates with clinical outcomes 82. lncRNA PVT1 promotes fatty acid β-oxidation by upregulating Mcl-1, which directly interacts with long-chain acyl coenzyme a dehydrogenase, while inducing MCL-1 to activate BAX and BAK, converting it from a monomeric protein to a mitochondrial outer membrane penetrating of oligomeric pores and promote apoptosis in ccRCC cells 83.

## LncRNAs and Amino acid metabolism

### LncRNAs as ceRNA

Arginine succinate synthase 1 (ASS1) is the main enzyme involved in endogenous arginine production, regulates the urea cycle and catalyzes the conversion of aspartate to urea-regulating enzymes. lncRNA 00312 is mainly located in the cytoplasm of ccRCC cells and may act as an competing endogenous RNA binding to MicroRNA, thus eliminating the repressive effect of MicroRNA on target gene transcripts, and lncRNA 00312 inhibits ccRCC proliferation and invasion and promotes apoptosis by downregulating ASS1 expression through the miR-34a-5p/ASS1 axis 84. Zhang B et al. found that LncRNA TUG1 is the ceRNA of Mir-141-3p and inhibits its expression, thereby down-regulating downstream β -catenin expression, activating C-MYC, accelerating glutamine metabolism of ccRCC cells, and thus promoting ccRCC migration 85.

### LncRNAs regulate amino acid-related metabolic pathways

In ccRCC cytoplasm, Wnt and mTOR signaling are associated with glutamine metabolism. β-linked proteins and the TCF family are key regulators of the Wnt signaling pathway, such as TCF-1, LEF-1 and TCF7L2, activate the downstream signaling target c-MYC, directly induce glutamine transporter protein ASCT2 expression to promote glutamine import and upregulate GLS both transcriptionally and post-transcriptionally to increase glutamine to glutamate conversion for subsequent oxidation in the TCA cycle 45. it's worth noting that MALAT1 regulates glutamine and glucose metabolism by upregulating SRSF1 expression enhanced translation of TCF7L2, while activating Wnt/β-catenin pathway to promote ccRCC malignant transformation process 86.

## LncRNAs as potential therapeutic targets

Currently, CT and histopathology are mostly used for clinical diagnosis of ccRCC; however, histopathological diagnosis is an invasive analysis and is not suitable for regular monitoring and assessment of disease progression. Recent studies have revealed that lncRNAs are involved in a variety of metabolic mechanisms or molecular signaling in ccRCC, regulating cancer cell genesis and development, providing a basis for clinical screening, early diagnosis and prognosis 44. Meanwhile, lncRNAs and related regulatory mechanisms are also potential targets for the treatment of ccRCC. For example, metformin. Adenine monophosphate activated protein kinase (AMPK) is a cellular energy sensor that reflects cellular energy status by undergoing phosphorylation and increasing activity when adenosine levels are elevated and adenosine triphosphate levels are decreased. Recently, metformin, an AMPK agonist, was shown to inhibit the metabolism and proliferation of ccRCC cells in several preclinical studies in ccRCC mouse models by Liu M et al. It is also suggested that metformin, under glucose-deficient conditions, promotes the transcription of genes related to cellular metabolism, such as c-Myc, by recruiting pyruvate kinase isozyme type M2 (PKM2) and β-linked proteins to form a complex through AMPK^87^. In addition, methyl tert butyl ether (MTBE) is associated with ccRCC cell proliferation, apoptosis and DNA damage. Zhao Y, et al. suggested that MET upregulates the expression levels of pro-apoptotic proteins such as Bax, Bak, caspase-3 and caspase-9 and downregulates the expression levels of Bcl-2 by activating reactive oxygen species, targeting the mitochondria-dependent apoptotic pathway and inducing DNA6 breakage which in turn affects ccRCC cell growth 88.

Glucose 6-phosphate dehydrogenase (6PGD) is an oxidative carboxylase, a component of the oxidative pentose phosphate pathway that plays an important role in the metabolic coordination of glycolysis, biosynthesis and proper redox state. Previous studies have shown that 6PGD expression is observed to be upregulated in several cancers, including liver, colon and breast cancers. Recent studies, for the first time, demonstrated that aberrant 6PGD expression is closely associated with ccRCC carcinogenesis, chemotherapy and immune resistance, revealing that 6PGD downregulation would activate AMPK signaling, leading to acetyl-CoA carboxylase 1 (ACC1) inhibition and reduced lipid synthesis, disrupting NADPH and NADH in ccRCC cells homeostasis *in vivo* and inhibits SIRT-1 activity, sensitizing ccRCC cells to general anticancer drugs 89. Therefore, 6PGD downregulation becomes a novel potential target for the treatment of ccRCC, which should be further explored and validated in conjunction with the clinic. In addition, N-Myc Downstream Regulated Gene 2 (NDRG2) was found to inhibit mTORC1 activity and synergize with mTOR inhibitors to inhibit glycolysis and glutamine catabolism in ccRCC cells 90.

## Conclusion

Metabolic reprogramming is critical in altering the biological behavior of ccRCC cells. Currently, lncRNAs are known to act as regulators of a variety of human malignancies and can directly or indirectly exert a broad and complex influence on ccRCC metabolic pathways and products, expanding our understanding of metabolic reprogramming in cancer. However, many key unknowns and limitations of studying the biological processes of LncRNAs still need to be addressed in further studies. First, how LncRNAs are exported from the nucleus to the cytoplasm, although most LncRNAs accumulate in the cytoplasm, how their localization or nuclear output is controlled remains unknown. Second, there is a balance between LncRNAs production, localization, and degradation, which is carefully regulated by chemical composition, trans-acting factors, and tumor microenvironment. When equilibrium is disrupted, the expression of LncRNAs changes. However, the specific mechanisms of biogenesis, distribution and degradation of LncRNAs remain unclear. Finally, studies on the metabolic regulation mechanism of lncRNAs in tumor cells are still incomplete, which results in limitations of relevant clinical treatments. Therefore, in this paper, we describe the different regulatory mechanisms or molecular signatures of LncRNAs regulating metabolic reprogramming in ccRCC cells. And it provides a basis for further research on the role and mechanism of lncRNAs in tumor metabolism, identification of new tumor markers and potential therapeutic targets in the future.

## Figures and Tables

**Table 1:** Related pathways / mechanisms of ccRCC metabolic reprogramming regulated by LncRNA.

LncRNAs	Metabolic pathway	mechanisms	Expression of ccRCC	Biological behavior of ccRCC	Reference
LncRNA SARCC	Glycolysis	miR-143-3p/AR/AKT;AR/HIF - 2/c - Myc signal	upregulation	Apoptosis	47,48
LncRNA KCNQ1DN	Glycolysis	cyclin D1/c-Myc/GLUTs	downregulating	Apoptosis	49
lncRNA ROR	Glycolysis	AUF1/c-Myc	upregulated	proliferation	50,51
LncRNA FILNC1	Glycolysis	AUF1/c-Myc	downregulating	Apoptosis	52
LncRNA THOR	Glycolysis	IGFBP-1/c-Myc/HK	upregulated	proliferation	56
LncRNA HOTAIR	Glycolysis	IGFBP2/GLUTs	upregulated	proliferation	57
LncRNA SNHG12	mitochondrial dynamics	miR-129-5p/MDM4/p53	upregulated	proliferation	59
LncRNA TP73-AS1	mitochondrial dynamics	mTORC/SREBP1/2;mTORC/SDH	upregulated	proliferation	61
IncRNA NDUFA4L2	mitochondrial dynamics	HIF-1		proliferation	64
IncRNA PANDAR	mitochondrial dynamics	NF-YA/PI3K/Akt/mTOR	upregulated	proliferation	66
LncRNA MEG3	mitochondrial dynamics	ST3Gal1/Bcl-2/prcaspase-2	upregulated	Apoptosis	68-70
LncRNA HOTAIR	mitochondrial dynamics	MICU1/Bcl-2/Mcl-1	upregulated	proliferation	71
LncRNA ITGB1	mitochondrial dynamics	Bcl-2/Mcl-1	upregulated	proliferation	73
LINC01094	lipid metabolism	miR-184/SLC2A;miR-224-5p/CHSY1	upregulated	proliferation	74,75
LncRNA TUG1	lipid metabolism	miR-31-5p/FLOT1	upregulated	proliferation	77
lncRNA DLX6-AS11	lipid metabolism	miR-26a/PTEN/PI3K-AKT	upregulated	Apoptosis	78
LncRNA AnxA3	lipid metabolism	aveolin1/FAs	downregulating	Apoptosis	79
LncRNA HAO2	lipid metabolism	LDs	downregulating	proliferation	80
LncRNA ALDH7A1	lipid metabolism	PPARα/FABP1/FAs	downregulating	Apoptosis	81,82
LncRNA PVT1	lipid metabolism	LCAD/FAO; MCL-1/BAX/BAK	upregulated	Apoptosis	84
lncRNA 00312	Amino acid metabolism	miR-34a-5p/ASS1	downregulating	Apoptosis	85
LncRNA TUG1	Amino acid metabolism	miR-141-3p/β-catenin/c-Myc	upregulated	proliferation	86
LncRNA MALAT1	Amino acid metabolism	SRSF1/TCF7L2/Wnt/β-catenin	upregulated	proliferation	87

## Abbreviations

RCC: Renal cell carcinoma
LncRNAs: Long-stranded non-coding ribonucleic acids
PDK: Pyruvate dehydrogenase kinase
GLUT-1: Glucose transporter 1
SGLT: Sodium glucose junction transporter
MCT1: Monocarboxylic acid transporter 1
G6PD: Glucose-6-phosphate dehydrogenase
MPC1: Mitochondrial pyruvate vector 1
ERR-α: Estrogen associated receptor α
IDH: Isocitrate dehydrogenase
METTL14: Methyltransferase-like 14
FAS: Fatty acid synthesis
LXRα: Liver X receptor α
FASN: Fatty acid synthase
SCD1: Stearoyl-coA desaturase-1
YB-1: Y box binding protein 1
LDs: Lipid droplets
CPT1A: Carnitine palmityl transferase 1A
PLIN2: Perisolipoprotein 2
MBOAT7: Membrane bound O-acyltransferase domain 7
HMGCR: 3-hydroxy-3-methylglutaryl-coenzyme A reductase
UBIAD1: Ectopic expression of the TERE1
SIRT4: Sirtuin 4
ICGC: International Cancer Genome Consortium
